# Transmission Success of Entomopathogenic Nematodes Used in Pest Control

**DOI:** 10.3390/insects9020072

**Published:** 2018-06-20

**Authors:** Sophie Labaude, Christine T. Griffin

**Affiliations:** Department of Biology, Maynooth University, W23 A023 Maynooth, Co. Kildare, Ireland; s.labaude@gmail.com

**Keywords:** entomopathogenic nematodes, Steinernematidae, Heterorhabditidae, infection, biocontrol

## Abstract

Entomopathogenic nematodes from the two genera *Steinernema* and *Heterorhabditis* are widely used as biological agents against various insect pests and represent a promising alternative to replace pesticides. Efficacy and biocontrol success can be enhanced through improved understanding of their biology and ecology. Many endogenous and environmental factors influence the survival of nematodes following application, as well as their transmission success to the target species. The aim of this paper is to give an overview of the major topics currently considered to affect transmission success of these biological control agents, including interactions with insects, plants and other members of the soil biota including conspecifics.

## 1. Introduction

Entomopathogenic nematodes (EPNs) represent a guild of soil-inhabiting nematodes capable of infecting a wide range of insects. Their free-living infective juveniles (IJs) penetrate insect hosts through natural openings or through the cuticle [1] and release their symbiotic bacteria into the hemocoel. The host is usually rapidly killed by the ensuing septicemia, and its cadaver is converted from insect to nematode biomass, mediated by the action of the bacteria in digesting host tissues into a nutritious soup for the nematodes. The bacteria also protect the cadaver resource against competitors, by producing bacteriocins, antimicrobials and other antibiotics [2,3]. IJs develop to adults and give rise to several successive generations until the resources become scarce. New IJs are produced and emerge from the cadaver, dispersing and seeking fresh hosts to infect. 

The two families Heterorhabditidae and Steinernematidae are widely studied, for two reasons: firstly, they both show great potential as inundative biological agents against numerous insect pests, in addition to or in replacement of chemical pesticides, and secondly, due to their short lifespan and ease of lab culture, they are increasingly used as model organisms in fundamental research into symbiosis and parasitism, inter alia. So far, about 90 species of Steinernematidae and 20 species of Heterorhabditidae have been described. However, only a handful of species are commercially produced for use in biological control [4], and these species (particularly *Steinernema carpocapsae, Steinernema feltiae* and *Heterorhabditis bacteriophora*) are also the ones that are most intensively researched [4,5,6,7,8]. Significant developments are being made towards increasing efficacy through strain selection and improved methods of production [9,10], formulation [11,12,13,14,15] and application [16,17,18,19]. Research on the biology and ecology of EPNs, such as mechanisms of infection and factors influencing their survival and behavior, underpins these developments and also helps make better predictions of their field performance. 

EPN transmission strategies have been shaped by natural selection to optimize fitness of the nematodes themselves, and are of intrinsic interest to parasitologists and evolutionary ecologists. In this paper, we review factors currently considered to impact EPN transmission success, particularly those that impact on biocontrol, with emphasis on recent developments and literature. 

## 2. Trait Diversity

**Figure 1:**
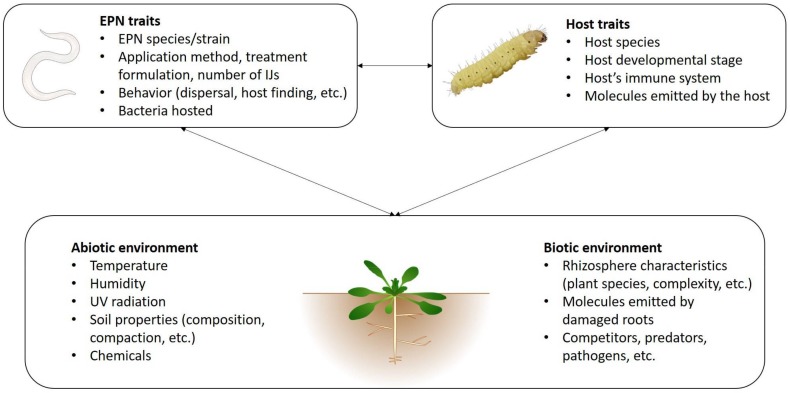
Factors influencing EPNs survival and efficacy.

Apart from some noticeable exceptions, such as *Steinernema scapterisci* which presents a narrow host range [20], most EPN species are capable of infecting a wide range of different insect species—at least when tested in the laboratory. However, the susceptibility of insect pests varies depending on EPN species. Although EPNs are already widely used in inundative biological pest control programs, a substantial part of current research effort concerns tests against additional insect pests, including groups as diverse as Dipteran, Lepidopteran, Isopteran, Hemipteran, Hymenopteran or Coleopteran species. Table 1 presents examples of recent studies (2014–present). Although some EPN species have been shown to be efficient against a large number of different pest species (e.g., *H. bacteriophora* or *S. carpocapsae*, see Table 1), several different species of nematodes are usually compared in order to identify the best species for the proposed purpose (e.g., [21,22,23,24,25]). Native species are often considered [23,25,26], as they are expected to be adapted to local conditions, ideally including the pest itself. Use of native species is also preferred for the purpose of limiting environmental risks. In addition, nematodes belonging to the same species but originating from different isolates can also drastically vary in their efficacy [27]. For instance, the mortality of *Bactrocera dorsalis* fruit flies induced by *Heterorhabditis taysearae* ranged from 51.2 to 96.1% depending on the isolate, despite all isolates originating from Benin [28]. Considering such important variation, finding the most efficient species/isolate is thus particularly important, and is quite a difficult task considering the large number of EPN species already described. Moreover, new species are regularly discovered. Within the five last years, at least two Heterorhabditidae and eight Steinernematidae have been described (Table 2), while already-known species were detected in new geographical locations [29,30]. More rarely, new species of bacteria are also described, such as *Photorhabdus heterorhabditis*, a symbiont of *Heterorhabditis zealandica* [31]. The laboratory screening step is of most use in rejecting species and strains with low virulence, but because many factors other than virulence are crucial to field success, it is advisable to bring more than one virulent strain to field testing. While screening different species and isolates is an important and practical step in a biocontrol program, the underlying reasons for species and strain specific differences in efficacy are rarely elucidated (Figure 1). These can include traits related to nematode behavior and virulence [32], while the bacterial symbiont is an important and often overlooked determinant of success of the nematode-bacterial complex. In the past, it was considered that each nematode species was associated with a particular symbiont species or subspecies [33], but it is becoming clear that certain species, particularly of *Heterorhabditis*, can associate with more than one symbiont (e.g., [34,35]). In nature, switching between symbionts with different properties can allow a nematode to effectively extend its niche. For instance, in desiccating conditions, *Heterorhabditis downesi* IJs had a higher reproductive success when they were associated with the bacteria *Photorhabdus temperata* subsp. *cinerea* compared to those associated with *P. temperata* subsp. *temperata* [35]. Reassociating nematodes with compatible but non-native symbionts is one possible route to strain improvement. However, any gain in virulence must be offset against a potential loss in other traits of the symbiosis, such as reproductive capacity [36]. *Steinernema feltiae* recombined with symbiont *Xenorhabdus bovienni* from different sources performed significantly better with the native symbiont and closely related strains than with those from more divergent sources [37]. 

Promising results from laboratory experiments on the efficacy of EPNs against insect pests frequently do not translate into success in field and greenhouse, as shown for example in tests of EPNs against the African black beetle *Heteronychus arator* [38] and the carob moth *Ectomyelois ceratoniae* [39].

This is not surprising, given the complexity of the soil environment with many interacting biotic and abiotic factors impacting nematode survival and host finding (Figure 1). Entomopathogenic nematodes used as biocontrol agents are usually applied in high numbers [40], but only a fraction of these succeed in finding a host. A dramatic decline in IJ numbers has been documented following the application of nematodes [41], which has been attributed to the detrimental effect of UV light and desiccation at the soil surface. Studies have shown inter-specific variation in nematodes’ tolerance to UV [42] and to desiccation [43,44,45,46,47]. Adequate environmental moisture is essential for IJ survival and movement [48,49,50]. Although it is often difficult to control this parameter in the field, one solution resides in the addition of adjuvants [21,26,51,52,53], in particular in the case of folial application of EPNs that induce a higher desiccation stress [54,55]. For instance, both the survival rates and infectivity of *Steinernema* species against two Lepidopteran pests were increased by the use of a surfactant in the nematode suspension [56]. Similarly, the negative effect of UV on nematodes can be reduced by using certain UV protectants [55]. In contrast, other application methods can also be used rather than nematode suspensions, such as nematode-infected cadavers [16] or pre-infected live insect hosts [19], both of which gave promising results. A more technically advanced alternative is the use of nematode-filled capsules. While lacking the natural IJ dispersal factors that may be present in insect cadavers [57,58], the use of capsules has the advantage of being able to include attractants and feeding stimulants for the target pest, and this approach has been successfully trialed against *Diabrotica virgifera virgifera* in maize [59]. Attracting the pest to the nematodes, rather than the reverse, reduces the importance of several of the key elements in the nematodes’ natural transmission strategy (dispersal, host finding, and many of the interactions with soil biota), relying more on virulence for the target pest and also compatibility with the formulation. 

Nematodes moving into the soil will be protected from UV and partly from desiccation, especially since IJs can adapt their vertical distribution according to the soil moisture and their own tolerance [60,61]. However, they remain vulnerable to other mortality factors, such as temperature extremes or predation, as well as starvation, resulting in a more gradual decline in their numbers over the weeks following the application. As for desiccation tolerance, nematodes have different thermal limits and optima depending on their species [43,44,62]. Temperature is probably the most important factor impacting EPN efficacy [23,25,38,48,51,63,64]. Temperature can also be used as a tool to improve EPN efficacy. For instance, conditioning *S. carpocapsae* and *Heterorhabditis megidis* for three weeks at 9 °C increased their efficacy against black vine weevils *Otiorhynchus sulcatus* [65]. 

## 3. Dispersal and Host Finding

The number of IJs exiting a host can range from tens of thousands to hundreds of thousands [101], while billions of them are applied to control pests [40]. We assume that each individual IJ has been shaped by natural selection to locate a suitable host in which to complete its life cycle, though strains may since have been artificially selected for improved biocontrol [102]. Even strains that have been bred for improved biocontrol performance and produced under conditions that are as uniform as possible consist of individual IJs, each with its unique history of gene x environment interactions [103]. IJs’ behavior changes as they age. The temporal change that receives most attention is the decline in “quality”, mostly measured as infectivity, as IJs age [104]. IJs are non-feeding and rely on a finite quantity of energy reserves that are critical for their survival and infection success. In particular, lipids and glycogen contents have been linked to infectivity in several nematode species [105,106,107]. To increase their chances of finding a suitable host, IJs employ a variety of strategies, and two types of model can be used to help conceptualize this [108]. Hierarchical models, developed for other parasites, divide the transmission process into a set of steps that may include dispersal, host-habitat location, host location and host acceptance, with each phase (phases are not necessarily distinct) characterized by responses to specific stimuli [108,109]. While each of these processes is studied in EPNs, the temporal phasing of them has received less attention, but we can assume that rapidly after their emergence from an insect host or their application as biocontrol agents, IJs move away in an initial dispersal phase characterized by random movements as well as potential responses to environmental signals that might bring them to their hosts’ habitat [109]. Dispersal of IJs is stimulated by factors associated with the spent cadaver [57]. Among the factors responsible are ascarosides, a diverse class of signaling molecules that regulate development and social behaviors in *Caenorhabditis elegans* and more widely among nematodes [58]. Ascaroside #9 accumulated in nematode-infected cadavers and in bioassays caused *S. feltiae* IJs to become more active and move away from the source location [58]. Once in the host’s habitat, nematodes rely on different types of signals, mostly chemicals [110] (although vibrations or temperature gradients might also be used [111,112,113]), bringing them to the area of the habitat that is modified by the presence of the host (host’s “active space”) [109], and ultimately to the host itself. 

A second conceptual model involves a distinction between EPN species as ambush or cruise foragers. *Heterorhabditis* species are characterized as cruisers, moving actively through the soil in search of their hosts, while the behavior of *Steinernema* species varies from cruisers to ambushers; ambushers mostly remain at the surface of the soil and lift their body into the air (nictation) or exhibit jumping behavior to attach to passing insects [114,115,116]. The behavior of *H. bacteriophora* and *S. carpocapsae* was recently studied in mesocosms [117]. In accordance with the cruiser-ambush theory, *H. bacteriophora* was more efficient at infecting non-mobile hosts (*Galleria mellonella* larvae maintained in cages) compared to mobile hosts, while the contrary was observed for *S. carpocapsae* [117]. Despite their foraging strategy, ambusher species also need to disperse. In another mesocosm experiment, a majority of *S. carpocapsae* were found to ambush near the source cadaver, while a majority of *H. bacteriophora* dispersed away from it [118]. However, around 4% of *S. carpocapsae* IJs exhibited a “sprinter” behavior, dispersing faster than the fastest *H. bacteriophora* and reaching 30–61 cm (in comparison, only 2% of *H. bacteriophora* IJs reached that far) [118]. The two different strategies are likely based on trade-offs with other parameters. Indeed, although *S. carpocapsae* responded promptly to the artificial selection of the fastest dispersers, the resulting enhanced dispersal was associated with reduced reproduction and nictation abilities [119]. The distinction between species based on foraging strategy should not be over-relied on for accurately predicting field success of a species. For example, *S. carpocapsae*, which is classified as an ambusher, was able to infect large pine weevils (*Hylobius abietis*) under the bark of tree roots as far as 30 cm deep in the soil [120]. In this and similar examples, the nematodes may be using tree roots as “routeways” to facilitate movement through soil.

Several factors are known to influence IJs dispersal, such as the vegetation [118,121] or the soil properties, in terms of composition and compaction [122]. Along with the presence of appropriate hosts, the impact of such factors on EPN survival and dispersal might explain the distribution of nematodes in habitats differing in plant communities or soil parameters [123,124]. Some insects can also serve as phoretic dispersers, such as the beetle *Calosoma granulatum*, recently shown to be capable of transporting *Heterorhabditis amazonensis* over long distances (over 40 cm) [125]. Recently, attention has been focused on the dispersal of IJs in groups, rather than as individuals [126,127]. Both *Heterorhabditis indica* (cruiser-type forager) and *S. carpocapsae* (ambusher-type forager) performed aggregative movement patterns, including in their dispersal phase [128]. Such group movements might increase the survival of IJs, through reduced evaporation rates in desiccating conditions, or through protection against predators (effect of dilution). It could also ensure a better infection success with the penetration of several IJs in a host, where the probability of finding a suitable mate would also be increased [128], though if nematodes regularly travel and mate with individuals from the same natal host, this should increase the risk of inbreeding. It is currently unknown to what extent group movement is a product of physical forces acting on the nematodes, or involves some form of chemical signaling. 

Eventually, the random dispersal of IJs might bring them into zones in which signals from the host or its habitat will be used for directing movements. In addition to the universally produced CO_2_ which is attractive for IJs of all EPN species [129], many specific host-derived odorants that stimulate host-seeking behavior by IJs have been identified and shown to be differentially attractive to different EPN species [110]. Recently it was shown that *Steinernema diaprepesi* and *H. indica* were attracted not only to beetle frass, but also to the sex pheromones of their weevil host [130]. Entomopathogenic nematodes widely use root-feeding insects as hosts. Consequently, stimuli initiating active search from IJs originate not only from the host itself, but also from roots, as indicators of likely host habitat, and more particularly damaged roots, indicative of the presence of the host. Although not specific to the host species, following a CO_2_ gradient is likely to bring nematodes into the rhizosphere, where potential hosts might then be detected based on their own chemicals or on chemicals emitted by the plant. While roots themselves are attractive, roots that are wounded by the feeding of herbivores emit volatiles or blends that specifically attract EPNs, a form of signaling that benefits both plants and nematodes [131,132]. This effect has been demonstrated for several species of plant, including recently for carrot, vine, fig and sugarcane [133,134,135]. In order to directly examine the foraging behavior of nematodes, Li et al. used pluronic gel, a transparent polymer allowing 3D observation of nematodes inside the gel. In accordance with previous results, they found that IJs preferentially aggregated around the wounded parts of roots [136]. In addition to the release of attractive compounds, roots might also provide nematodes with physical “routeways”, facilitating their progression into the soil [137]. Moreover, the complexity of the root architecture can interact with the herbivore-induced plant volatiles to impact the foraging behavior of nematodes. Using artificial model-roots with different degrees of complexity and connectivity, Demarta et al. found that host-finding by *H. megidis* was facilitated by low root complexity. However, the addition of a synthetic root volatile, (E)-β-caryophyllene, changed this pattern and favored the nematodes foraging on the most complex model-roots [138]. The effect of (E)-β-caryophyllene on the behavior of *H. megidis* was also found to depend on the type of soil, while the diffusion of the compound in the soil depended on its humidity [139]. Aboveground stimulation can also influence the recruitment of EPNs in the soil. In particular, the stimulation of the salicylic acid pathway, a plant defense pathway, led to the attraction of *S. diaprepesi* nematodes in the absence of their hosts [50]. Filgueiras et al. also found a strong effect of elicitors of plant defense applied on leaves of corn seedlings on the recruitment of *H. amazonensis* nematodes and suggested that treating crops with elicitors might be a good strategy to increase the success of the biological control of EPNs [140].

Recent evidence suggests that the response of nematodes to volatile compounds such as (E)-β-caryophyllene or pinene could be modified by their experience (previous exposure to the compound) [141]. Intriguingly, the behavior of *H. indica* individuals that were not previously exposed to α-pinene depended on interspecific relationships; although naive *H. indica* alone were repelled by α-pinene, they exhibited a preference for this compound when they were associated to previously-exposed *S. diaprepesi*, [141].

## 4. Infection

Having located a host, IJs may choose to infect or not, depending on its suitability. Since IJs of different species respond differently to different species of insect [110], some amount of host selection may already be accomplished during the host-seeking phase. Apart from some specialist species [20], most EPNs can infect a wide range of different host species, at various developmental stages, with substantial differences in susceptibility to nematodes both between species and between developmental stages of the same species. Insect larvae and pupae are often found to be more susceptible to EPN infection than adults [25,85,142]. Such differences in susceptibility can result from various mechanisms, such as differences in behavior (in particular higher activity levels and avoidance behaviors in adults), immune system or physical barriers to nematode penetration. For instance, the low susceptibility of certain pupae, which was also observed in other studies [49,77], could be due to a lack of natural entry routes for nematodes, as well as a tougher cuticle [76]. Susceptibility can also vary among different larval instars, as was observed in armyworms and mosquitoes [72,88]. Interestingly, large pine weevils (*H. abietis*) infected by *H. downesi* or *S. carpocapsae* as pupae died as adults, suggesting that IJs can infect pupae, survive through their host metamorphosis and kill the adults [142]. In social insects, variation in susceptibility was observed among different castes. While *Steinernema karii* induced a higher mortality in workers of the termite species *Coptotermes formosanus* compared to soldiers [82], the opposite effect was observed for the two other termite species *Macrotermes bellicosus* and *Trinervitermes occidentalis* exposed to *H. indica* and *Heterorhabditis sonorensi* [83]. Finally, although EPNs are typically thought of as infecting and killing live insect hosts, IJs can also act as scavengers. For instance, although their progeny was lower than that recorded in live hosts, both *Steinernema kraussei* and *H. megidis* were able to reproduce in freeze-killed *G. mellonella* larvae [143]. The ability to utilize additional resources for reproduction may facilitate persistence of EPN populations in soil post application. 

Differences in the success of nematode infection documented above can be partly due to the response of the host’s immune system to the nematode-bacteria complex. The immune response of insects to EPNs and the mechanisms used by nematodes and bacteria to evade or defeat it have been extensively studied in recent years (see the review by Eleftherianos et al. [144]). Following the penetration of IJs into the insect host through natural openings and their establishment in the hemolymph, the IJs then release their symbiotic bacteria. These are either from the *Photorhabdus* genus for *Heterorhabditis* species, or from the *Xenorhabdus* genus for *Steinernema* nematodes. The subsequent release of toxic and immunosuppressive compounds by the bacteria cause death of the host by septicemia [145,146]. Axenic nematodes, especially *Steinernema* species, are also capable of causing the death of their hosts [146,147], in particular through the release of a venom containing a high proportion of proteases (serine carboxypeptidases, trypsins, eukaryotic aspartyl proteases, zinc carboxypeptidases) and protease inhibitors [147]. However, the association between nematodes and bacteria is important in the virulence of the complex [148]. In an experiment where nematodes from different *Steinernema* species were isolated from their symbiotic bacteria, combinations of nematodes with “foreign” bacteria resulted in a reduction of the virulence of the complex against *G. mellonella* larvae, as well as a reduction in nematode progeny [149].

Following host penetration, the release of bacteria by nematodes is usually delayed in the host by 30 min for *Heterorhabditis* species and several hours for *Steinernema* nematodes [150]. There is thus a possibility for the insect to neutralize its parasite before the bacterial challenge. Insects’ reactions to nematode infection include the melanization and encapsulation of the parasites with hemocytes. Many immune factors have been shown to vary in the hemolymph of the host following the entry of nematodes, including both humoral and cellular responses. For instance, many studies documented modifications through time in the hemocyte counts [151,152,153] and the enzyme activity of the host such as proteases and phenoloxidase [152,153,154,155]. Nematodes and their symbiotic bacteria can both inhibit their hosts’ immune system, suppressing the melanization process and depressing the antimicrobial response [146,153,156]. Since IJs are applied in high numbers, it is likely that at least some hosts get infected by numerous nematodes. Such multiple infections, although leading to competition between nematodes (see below), lower the risk of them being overcome by the host’s immune system. However, Kenney and Eleftherianos suggested that the success of infection of EPNs used in biocontrol programs could be improved by developing nematode-bacteria complexes that produce proteins necessary for evasion of the host’s immune system, or nematodes with a short delay in the ejection of their bacterial symbionts [157]. 

## 5. Interaction with the Biotic and Abiotic Environment

As shown above, environmental parameters such as temperature, humidity, types of vegetation and soil properties can affect nematode survival and virulence, while infection also depends on complex interactions between IJs, their symbiotic bacteria and the host (Figure 1). However, other abiotic parameters and biotic interactions are particularly worthy of interest, especially when considering nematodes as biocontrol agents. 

Nematodes applied inundatively to soil can affect other species, including non-target hosts or potential competitors such as indigenous nematodes [158]. Studies investigating the environmental risks linked to the use of EPNs generally re-affirm the low impact of this form of biocontrol on non-target species [159]. For instance, non-target species were unaffected by the use of *H. bacteriophora* applied against corn rootworm larvae in maize [160]. The effect on non-target species will depend on the biology of these species, and laboratory studies can help identify susceptible species and formulate recommendations for risk reduction, if considered necessary [161]. Special consideration needs to be given to ecologically important species, and potential impact on their roles in ecosystems. However, as for tests against target species, laboratory tests of susceptibility of important non-targets may not accurately predict impact in the field. Recently, bumble bees (*Bombus terrestris*) were reported to suffer 80% mortality when exposed in the laboratory to soil treated with EPNs at recommended field concentrations [162]. Moreover, IJs were able to proliferate in the bee cadavers, which the authors suggested may represent a potential threat for other members of their colony [162]. However, factors such as the preferred habitat for bumble bee nests, the depth and structure of the nests, and behavior of infected individuals [163] or their hygienic colony mates [164], will likely present significant ecological barriers to EPN infection of bumble bees. Safety may also be examined in terms of persistence of EPNs in the treated area. In a field experiment, *S. carpocapsae* was detected in the soil up to two years after application, but not after four years, and its presence was positively correlated with that of its large pine weevil host [165], an optimistic result regarding potential environmental risk. 

EPNs used for biocontrol are also likely to interact with other organisms, especially those belonging to the trophic network of their hosts. Within the few days following infection, host cadavers release scavenger deterrent factors (SDF), chemical compounds produced by nematodes’ symbiotic bacteria that deter potential scavengers and thus protect the host cadaver [166]. A wide range of species, including various arthropods [166,167] and birds [168], are known to be sensitive to such factors, avoiding infected cadavers or promptly rejecting them after an attempt to consume them. Recently, laboratory tests on three cyprinid fish species showed they were also sensitive to SDFs, rejecting *G. mellonella* larvae killed by *Heterorhabditis* or *Steinernema* species, and preferentially feeding on freeze-killed mosquito (*Aedes aegypti*) larvae compared to nematode-killed ones [169]. Tests on the carabid beetle *Carabus granulatum*, a predator of the lepidopteran host of *H. amazonensis*, showed that larvae and adults both avoided feeding on infected larvae when they had a choice [170]. In forced feeding experiments, the consumption of infected larvae led to a high mortality of the predator [170]. However, not all insects are repelled by nematode-infected hosts. For instance, while crickets and springtails avoided *Steinernema*-killed insects, consumption was observed for ants, cockroaches, mites and earwigs [171]. In addition, springtails *Sinella curviseta* and *Folsomia candida* were found to directly consume IJs [171]. As suggested by the authors, the scavenger and predator effect of soil arthropods could have a top-down regulatory impact on nematodes. 

Nematodes can also be in direct competition with other organisms. In the case of the carabid *C. granulatum* cited above [170], the predator is not only a threat for the integrity of the host cadaver, and thus for the reproduction success of nematodes; IJs and beetles compete for the same resources, that is, living prey or hosts. Consequently, the inundative use of EPNs could decrease the number of insect prey available for predators, with consequences on their populations. Parasitoid insects also compete for the same resource, as well as being susceptible to infection by nematodes themselves. Despite negative interactions demonstrated in the laboratory, this is less often documented in the field [172,173]. For example, in field trials, the use of nematodes against Caribbean fruit flies *Anastrepha suspense* did not reduce the density of their emerging parasitoid *Diachasmimorpha longicaudata* [174]. The most direct competitors of EPNs might be nematodes themselves, either indigenous entomopathogenic nematodes or free-living nematodes (FLN). For instance, FLNs from the genus *Oscheius* show scavenger behavior and are capable of reproducing in freeze-killed as well as already infected insect cadavers [175]. Competition with *Oscheius* species in host cadavers resulted in a substantial reduction in the number of EPN progeny, with inter-specific differences in their ability to overcome intraguild competition [175,176]. A more recent study found contradictory results with no evidence of competition between two *Oscheius* species and EPNs [143]. Apart from competition for resources, a direct interspecific killing behavior has also been documented among four *Steinernema* species, as well as intra-specific killing among males [177,178]. Because of their negative interaction on other nematodes, EPNs can be used to control pest species. Many studies already documented the efficacy of EPNs against pest nematodes (reviewed by Kenney and Eleftherianos [157]). For instance, several species of EPNs, and particularly their bacteria, proved to be efficient against the root-knot nematode *Meloidogyne incognita*, a parasite of tomato plants [179,180,181]. More complex and unexpected interactions between EPNs or their bacteria and other organisms are reported, such as the induction by EPNs of systemic resistance in tomatoes against insect pests and bacterial diseases [182].

The plant host can also affect the susceptibility of insects feeding on it. For instance, *Steinernema riobrave* nematodes showed a reduced virulence and reproduction on *Helicoverpa zea* larvae reared on tobacco plants compared to those feeding on tomato and eggplants [183]. There is an increasing use of GMO plants in crops, many of them producing their own insecticidal proteins. In a recent study, no significant effects on the virulence, reproduction and host preference of *H. bacteriophora* were found when diamondback moth (*Plutella xylostella*) was reared on broccoli leaves genetically modified to produce CrylAc proteins [184]. Additional levels continue to be added to the inter-organismal interactions in which EPNs are implicated—most recently, the insect host’s endosymbionts. For example, the presence of *Wolbachia* endosymbionts promoted the survival of *Drosophila melanogaster* infected with *S. carpocapsae* through effects on the insect’s immune response [185]. 

## 6. Conclusions and Future Directions

The use of EPNs against pests is particularly timely given the negative impact of the use of chemical pesticides. Applied research largely focuses on improving their use on a large scale, through improved nematode culture methods, storage, treatment formulations and application methods. Spreading billions of nematodes in places where they are not always native represents a potential environmental risk that deserves attention. In this respect, long-term studies combining laboratory experiments followed by field tests are particularly relevant. In addition, a better understanding of the biology and ecology of EPNs is likely to give new directions in their use against pests. It is now getting easier to access large datasets of information through proteomic and transcriptomic studies. For instance, such approaches were used to describe nematode infection. The venom proteome of *S. carpocapsae* IJs was recently analyzed using mass spectrometry, highlighting the presence of 472 venom proteins including numerous proteases and protease inhibitors, as well as toxin-related proteins potentially linked to the suppression of host immune system [147]. In addition, the host immune response could be better understood with transcriptomic analyses following nematodes infection [186,187,188]. Genetic analyses, including sequencing, are particularly useful to better understand the diversity of nematodes [189,190] and their symbiotic bacteria [191,192], and to study their phylogenetic relationships [193]. Such phylogenies can help understand the evolution of certain traits. For instance, Blackburn et al. reconstituted the phylogenetic relationships of 18 strains of *Photorhabdus* and also tested their virulence against two hosts. Results showed a general evolutionary trend towards an increase in virulence in *Photorhabdus* [194]. Finally, genomic and proteomic data provide a large number of genes that are potential candidates for the improvement of EPNs, from the survival of IJs in certain conditions to an increase transmission and killing efficacy. Although artificial selection solely based on phenotypic traits can lead to improved nematodes [195], understanding the mechanisms might lead to better efficacy, especially on traits where artificial selection is complicated [196].

## Figures and Tables

**Table 1:** Recent studies (2014—present) investigating the effect of entomopathogenic nematodes against insect pests.

Pest group	Pest Species	Nematode Species Tested	Reference
Coleoptera	*Anomala graueri* (white grub)	*H. bacteriophora, S. carpocapsae, S. longicaudum*	Kajuga et al., 2018 [66]
	*Curculio elephas* (chestnut weevil)	*H. bacteriophora, S. glaseri, S. weiseri*	Demir et al., 2015 [67]
	*Holotrichia oblita* (white grub)	*H. bacteriophora, S. longicaudum*	Guo et al., 2015 [68]
	*Hylobius abietis* (large pine weevil)	*S. carpocapsae, S. downesi*	Kapranas et al., 2016 [69]
	*Phyllotreta cruciferae* (crucifer flea beetle)	*H. bacteriophora, H. indica, S. carpocapsae, S. feltiae*	Antwi and Reddy 2016 [70]
	*Polyphylla fullo*	*H. bacteriophora, S. glaseri, S. weiseri*	Demir et al., 2015 [67]
	*Rhynchophorus ferrugineus* (red palm weevil)	*H. bacteriophora, S. carpocapsae, S. feltiae*	Manzoor et al., 2017 [24]
	*Strategus aloeus* (oil palm chiza)	*H. bacteriophora, H. indica, S. colombiense, S. feltiae, S. websteri*	Gómez and Sáenz-Aponte 2015 [71]
Diptera	*Aedes aegypti* (yellow fever mosquito)	*H. baujardi, S. carpocapsae, H. indica,*	Cardoso et al., 2015 [72]
	*Bactrocera dorsalis*	*H. indica, H. tayserae*	Godjo et al., 2018 [28]
	*Bactrocera tryoni* (Queensland fruit fly)	*H. bacteriophora, S. carpocapsae, S. feltiae*	Langford et al., 2014 [49]
	*Bradysia odoriphaga* (chive maggot)	*H. bacteriophora, S. carpocapsae, S. feltiae, H. indica, S. longicaudum*	Bai et al., 2016; Wu et al., 2017 [16,73]
	*Chironomus plumosus*	*H. bacteriophora, S. carpocapsae, S. feltiae, S. kraussei*	Edmunds et al., 2017 [74]
	*Drosophila suzukii* (spotted wing drosophila)	*H. bacteriophora, S. carpocapsae, S. feltiae, S. kraussei*	Cuthbertson and Audsley 2016; Hübner et al., 2017; Garriga et al., 2018 [75,76,77]
	*Musca domestica* (housefly)	*H. indica, S. abbasi, S. carpocapsae, S. feltiae, S. glaseri*	Archana et al., 2017 [78]
	*Rhagoletis cerasi* (European cherry fruit fly)	*H. bacteriophora, H. marelatus, S. carpocapsae, S. feltiae*	Kepenekci et al., 2015 [23]
	*Stomoxys calcitrans* (stable fly)	*H. bacteriophora, H. baujardi*	Leal et al. 2017 [79]
Hemiptera	*Eriosoma lanigerum* (wooly apple aphid)	*H. bacteriophora, H. megidis, S. carpocapsae, S. feltiae, S. glaseri, S. kraussei*	Berkvens et al., 2014 [80]
	*Planococcus ficus* (vine mealybug)	*S. yirgalemense*	Le Vieux and Malan 2013 [81]
	*Trialeurodes vaporariorum* (greenhouse whitefly)	*H. bacteriophora, S. feltiae*	Rezaei et al., 2015 [21]
Hymenoptera	*Cephus cinctus* (wheat stem sawfly)	*H. bacteriophora, H. indica, S. carpocapsae, S. feltiae, S. glaseri, S. kraussei, S. riobrave*	Portman et al., 2016 [52]
Isoptera	*Coptotermes formosanus* (formosan subterranean termite)	*S. karii*	Wagutu et al., 2017 [82]
	*Macrotermes bellicosus* (termite)	*H. indica, H. sonorensi*	Zadji et al., 2014 [83]
	*Trinervitermes occidentalis* (termite)	*H. indica, H. sonorensi*	Zadji et al., 2014 [83]
Lepidoptera	*Cydia pomonella* (codling moth)	*H. bacteriophora, S. feltiae, S. jeffreyense, S. yirgalemense*	Odendaal et al., 2016 [25,26,84]
	*Ectomyelois ceratoniae* (carob moth)	*H. bacteriophora, S. carpocapsae, S. feltiae*	Memari et al., 2016 [39]
	*Ephestia kuehniella* (mill moth)	*S. carpocapsae, S. feltiae, S. riobrave*	Ramos-Rodríguez et al., 2006 [85]
	*Heliothis subflexa*	*H. bacteriophora, S. carpocapsae, S. feltiae, S. websteri*	Bolaños et al. 2016 [86]
	*Paranthrene diaphana* (clearwing moth)	*H. bacteriophora, S. carpocapsae, S. feltiae*	Azarnia et al., 2018 [22]
	*Plodia interpunctella* (Indian meal moth)	*S. riobrave, S. feltiae, S. carpocapsae*	Ramos-Rodríguez et al., 2006 [85]
	*Plutella xylostella* (diamondblack moth)	*S. carpocapsae*	Sunanda et al., 2014 [87]
	*Spodoptera litura* (tobacco cutworm)	*H. bacteriophora, S. glaseri*	Safdar et al., 2018 [88]
	*Synanthedon exitiosa* (peachtree borer)	*S. carpocapsae*	Shapiro-Ilan et al., 2016 [17]
	*Thaumetopoea wilkinsoni* (pine processionary moth)	*S. affine, S. feltiae*	Karabörklü et al., 2015 [89]
	*Tuta absoluta* (tomato leaf miner)	*H. bacteriophora, S. carpocapsae, S. feltiae*	Van Damme et al., 2016; Kamali et al., 2018 [51,63]
	*Zeuzera pyrina* (leopard moth)	*H. bacteriophora, S. carpocapsae*	Salari et al., 2015 [90]

**Table 2:** Recently discovered species of entomopathogenic nematodes.

New Species	Place	Reference
*Heterorhabditis noenieputensis*	South Africa	Malan et al., 2014 [91]
*Heterorhabditis pakistanense*	Pakistan	Shahina et al., 2017 [92]
*Steinernema balochiense*	Pakistan	Fayyaz et al., 2015 [93]
*Steinernema beitlechemi*	South Africa	Çimen et al., 2016 [94]
*Steinernema biddulphi*	South Africa	Çimen et al., 2016 [95]
*Steinernema innovation*	South Africa	Çimen et al., 2015 [96]
*Steinernema poinari*	Czech Republic	Mráček and Nermuť 2014 [97]
*Steinernema pwaniensis*	Tanzania	Půža et al., 2017 [98]
*Steinernema tbilisiensis*	Georgia	Gorgadze et al., 2015 [99]
*Steinernema tophus*	South Africa	Stock 2014 [100]
